# Incubation time effect on releasing available phosphorus in saline sandy soil as a function of bone char application

**DOI:** 10.1038/s41598-025-14997-8

**Published:** 2025-08-12

**Authors:** Abu El-Eyuoon Abu Zied Amin

**Affiliations:** Soils and Water Department, Faculty of Agriculture, Assiut University, P.O. Box: 71526, Assiut, Egypt

**Keywords:** Bone char, Incubation time, Phosphorus availability, Phosphorus fractionation, Saline sandy soil, Soluble sulfate, Environmental chemistry, Chemistry

## Abstract

To face the current crisis in global fertilizer prices, especially in developing countries where their food security has been greatly affected, alternative sources must be found for phosphate fertilizers, whose main source is phosphate rock, which is non-renewable and subject to depletion. Therefore, this study aims to evaluate the effect of the incubation period on the availability and fractionation of phosphorus in saline sandy soil under bone char addition. About 100 g of soil was placed in an airtight plastic jar and mixed thoroughly by adding 0.4 g of bone char. This experiment was incubated for 7, 16, 35, 65, and 84 days. The results obtained from this study revealed a significant increase (*p* ≤ 0.01) in available phosphorus with applying bone char in saline soil after 7, 16, and 35 days of incubation compared to the initial soil (before the incubation and unamended). Relative to the initial soil, the concentration of available phosphorus increased by 33.7%, 19.5%, and 12.3% after 7, 16, and 35 days, respectively. The results showed that increasing the incubation time significantly decreased phosphorus availability in saline soil after bone char application. The NaHCO_3_-Pi, HCl-Pi, and Res-Pi fractions increased significantly with the addition of bone char to the soil under study at all incubation periods compared to the initial soil. Inorganic phosphorus fractions after bone char application to saline sandy soil followed the order of HCl-Pi > Res-P > NaHCO3-Pi > NaOH-Pi > NH_4_Cl-Pi. In this context, these findings concluded that bone char amendment could be a potential P-source for agriculture in saline sandy soils to confront the high prices of phosphate fertilizers.

## Introduction

Soil salinization seriously threatens the productivity of global agriculture, ecosystem stability, and resource sustainability^1^. A soil salinization process can be classified into two categories: (1) primary salinization is attributed to natural dynamics such as hydrology, natural drought, aeolian salt deposition, and parent rock materials^2,3^ and (2) secondary salinization results from anthropogenic activities such as irrigation, road salting, and fertilization. Both primary and secondary salinization are expected to increase under climate change^3^. Approximately 1–2 million hectares of soil are salinized each year globally, affecting much of the crop production and making the soil unusable for farming^4,5^. Hence, managing salt-affected soils is essential in cultivating these soils^6^. Sustainable management of salt-affected soils is critical to meeting food grain production for an ever-rising population in the world. The low productivity of saline soil is not only attributed to salt toxicity or damage caused by excess amounts of soluble salts, but also arises from the lack of available nutrients, especially nitrogen and phosphorus, as well as organic matter content^7^.

Phosphorus is usually present in rock formations and ocean sediments in the form of phosphate salts and is released through weathering into the soil solution to be taken up by plants^8^. Moreover, phosphate rock is a non-renewable source of phosphorus and at the same time the main source for the production of phosphate fertilizers in the world^9^. Phosphorus is considered one of the most limiting macronutrients for crop productivity, and phosphorus deficiency is a common phenomenon in soils worldwide^10^. Phosphorus is a crucial nutrient for plant growth, and its effectiveness significantly impacts plant growth. Phosphorus availability in the soil is greatly influenced by many factors such as soil salinity, soil pH, and soil alkaline phosphatase activity^11^. Soil salinity is one of the most important factors affecting the availability of phosphorus in the soil, as it affects the surrounding environment^10,12^.

With the growing demand for agricultural production and the possibility of global production peaking in the coming decades, increasing attention is being paid to phosphorus as a non-renewable resource^13^. Since 2020, a pandemic, geopolitical conflicts, trade wars, and rising fuel prices have caused phosphorus commodity prices up more than 400%, contributing to the current food crisis. The current fertilizer price crisis is a wake-up call for the international community to address the global phosphorus challenge^14^. Moreover, the demand for global food security has contributed to significant development in the food production and slaughtering industries, particularly bone, which represents a great load on the companies to recycle and manage wastes and by-products^15^. Reusing waste or other alternative sources of phosphorus is an option to solve the future phosphate fertilizer supply problem. Alternative sources can be used to reduce reliance on mining to produce phosphate fertilizers. Animal bones are converted into bone char through the process of pyrolysis, which in turn can be used as a source of phosphorus^16^. Bone char is a material primarily composed of 70–76% hydroxyapatite, 9–11% carbon, 7–9% calcium carbonate, acid-soluble ash < 3%, calcium sulfate 0.1–0.2%, and iron as Fe_2_O_3_ < 0.3%^17^. Bone char is characterized by being rich in phosphorus and having a relatively large specific surface area. Thence, if added to soil, they likely contribute to adsorption and desorption processes of ions, organic molecules, and colloidal substances, which in turn leads to improving soil fertility^17^. Using bone char as a fertilizer is a promising alternative to phosphate fertilizers^18^. The dissolution of phosphorus from bone char to the soil solution is highly dependent on soil chemical properties^19,20^. Therefore, the international community needs to search for renewable sources of phosphorus, and here today we are in the process of using a renewable source of phosphorus, which is bone char. This study is one of the few that examines the behavior of bone char in saline soil and its effect on the release of available phosphorus and its fractionation, given the widespread presence of saline soil around the world, which is a result of climate change, consequently affecting the agricultural production of this soil at present. Also, there is very little systematic information available on the release and fractionation of phosphorus in saline soil amended with bone char. One of the priorities of this study was to find a renewable source of phosphate fertilizers in the form of bone char, which is produced by recycling slaughterhouse waste like bones. Therefore, this study aims to evaluate the effect of the incubation period on changes in some soil chemical properties and availability and fractionation of phosphorus in saline sandy soil by applying bone char. 

## Materials and methods

### Bone char preparation and its chemical analysis

The bone char used in this study was prepared from cow bones collected from home slaughtering in rural areas of Assiut Governorate, Assiut, Egypt. Before pyrolysis, the fat, marrow, and gelatin present in the bones were removed by boiling them in water several times. After this step was completed, the bones were scraped with a knife to remove any tissues and adhering materials, and then broken into small pieces of 15 to 25 cm. The broken bones were placed in a metal container and covered well with aluminum foil to reduce the amount of oxygen that could enter them. After that, they were pyrolyzed in a limited oxygen environment using a muffle furnace. The pyrolysis conditions of bone char production were performed by pyrolyzing at 650 °C with increasing pyrolysis temperature by 10 °C min^− 1^ (heating rate), and the residence time was two hours. After the pyrolysis process was completed, the resulting bone char was cooled at room temperature and ground in a stainless-steel mill, and passed through a 1 mm sieve. The pH of produced bone char was measured in a 1:2.5 ratio of bone char in distilled water suspension by a glass electrode^21^. Bone char extract prepared at a ratio of 1:5 cow bone char (g)/distilled water (ml) after 30 min end-over-end shaking and 1 h standing. The mixtures are then filtered using filter paper. Electrical conductivity (EC) was measured in the extracts using an electrical conductivity meter^21^. The soluble calcium in bone char extract was estimated by titration using Na_2_EDTA (Ethylenediaminetetraacetic acid disodium) solution^22^. Available phosphorus in bone char was extracted by 0.5 M NaHCO_3_ (pH 8.5) according to Olsen et al. (1954)^23^. The total content of phosphorus and calcium in bone char was digested by a mixture of concentrated acids of sulfuric, nitric, and perchloric (Grimshaw, 1989)^24^. The phosphorus in all extracts is determined colorimetrically using the chlorostannous phosphomolybdic acid method (Jackson, 1973)^21^. The important chemical properties of the produced bone char are shown in Table 1 according to Amin^25^.

**Table 1:** Some important chemical characteristics of the bone char used in this study according to Amin^25^. (All data are presented as the mean ± the standard error (SE)).

Soil property	Unit	Value ± SE
pH (1:2.5)		8.25 ± 0.01
EC (1:5)	dS m^− 1^	3.12 ± 0.00
Soluble calcium (1:5)	mg kg^− 1^	101.67 ± 2.48
Soluble phosphorus (1:5)	mg kg^− 1^	4.72 ± 0.01
Available phosphorus (Olsen-P)	mg kg^− 1^	1552.42 ± 23.90
Total phosphorus	g kg^− 1^	140.78 ± 0.04
Total calcium	g kg^− 1^	288.45 ± 1.88

*EC* electrical conductivity.

### The incubation experimental design

The incubation experiment was conducted in the Soil Chemistry Laboratory of the Soils and Water Department, Faculty of Agriculture, Assiut University. In the present study, the saline soil was collected at a 0–20 cm depth in Wadi El-Assiuty, Assiut, Egypt. The Saline soil under study was air-dried, sieved to pass through a 2-mm sieve, and kept for the performance of an experiment. The main physical and chemical properties of the soil under study are presented in Table 2. About 100 g of soil was placed in an airtight plastic jar (330 ml) and mixed thoroughly with 0.4 g of added bone char (equivalent to approximately 563 mg P kg^− 1^ soil based on the total P in bone char). The water content in the soil in all jars was adjusted to 50% of the saturation capacity via distilled water. The incubation experiment was laid out in a completely randomized design with three replicates and incubated for 7, 16, 35, 65, and 84 days under 23 °C in the dark. Jars are opened from time to time to preserve aerobic conditions and soil moisture content by weighing the jars and adding distilled water equivalent to the water loss. After every incubation period, the whole soil in the jar is taken, air-dried, and then crushed well with thorough mixing to increase the homogeneity of the sample. Then, stored to perform soil chemical analysis.

**Table 2:** Physicochemical properties of the soil used in this study (All data are presented as the mean ± the standard error (SE)).

Property	Unit	Value ± SE
Sand	g kg^− 1^	89.2 ± 0.00
Silt	g kg^− 1^	5.00 ± 0.20
Clay	g kg^− 1^	5.80 ± 0.20
Texture		Sand
OC	g kg^− 1^	1.61 ± 0.04
CaCO_3_	g kg^− 1^	160.49 ± 3.08
ACaCO3	g kg^− 1^	0.00
pH		8.05 ± 0.01
EC	dS m^− 1^	7.07 ± 0.02
Soluble Ca	mmol kg^− 1^	46.50 ± 0.14
Soluble Mg	mmol kg^− 1^	6.34 ± 0.21
Soluble Na	mmol kg^− 1^	108.03 ± 0.89
Soluble HCO_3_^−^	mmol kg^− 1^	5.83 ± 0.64
Soluble Cl^−^	mmol kg^− 1^	122.55 ± 0.82
Soluble SO_4_^2−^	mmol kg^− 1^	58.64 ± 0.62
Available phosphorus	mg kg^− 1^	6.91 ± 0.05

*OC* organic carbon, *EC* electrical conductivity, *CaCO*_3_ calcium carbonate, *ACaCO*_3_ active calcium carbonate.

### Soil chemical analysis

Soil pH was estimated in distilled water suspension (1:1) via a glass electrode. Soil extracts were prepared at a ratio of 1:2.5 soil (g)/distilled water (ml) after end-over-end shaking for 1 h. Electrical conductivity (EC) was measured in the soil extracts using an electrical conductivity meter^21^. Soluble calcium and magnesium in the soil extracts were estimated by titration with Na_2_EDTA solution (disodium ethylene diamine tetra-acetic acid); soluble Na was analyzed by the flame photometry method. Soluble bicarbonate (HCO_3_) + carbonate (CO_3_) was titrimetrically determined using HCl acid, soluble sulfate was estimated by the turbidimetry method using barium chloride^22^and soluble chloride was determined using silver nitrate solution^21^. The available phosphorus (Olsen-P), in soil samples, was extracted by 0.5 M NaHCO_3_ at pH 8.5 according to Olsen et al.^23^. The phosphorus in all extracts was measured by a spectrophotometer using the chlorostannous phosphomolybdic acid method in a sulfuric acid system^21^.

### Inorganic phosphorus sequential extraction

Inorganic phosphorus fractions in the soil samples were carried out by the sequential chemical extraction method reported by Hedley et al.^26^ and modified by Chen et al.^27^. A 2.50 g of air-dried soil was put into 100 ml plastic tubes. Initially, 50 ml of 1 M NH_4_Cl was added to the soil in tubes and shaken for 16 h, followed by centrifugation (4000 rpm), and the supernatant was collected. 50 ml of 0.5 M NaHCO_3_ was added to the remained soil from the previous step, followed by shaking and a centrifugation process again. Then, 50 ml of 0.1 M NaOH was added to the remaining soil and shaken for 16 h, followed by centrifugation as before. After that, 50 ml of 1 M HCl was added to the remaining soil and shaken for 16 h, followed by centrifugation as before. Finally, the remaining soil was digested by a mixture of concentrated acids such as sulfuric, nitric, and perchloric. Phosphorus in the supernatants was analyzed colorimetrically using the chlorostannous phosphomolybdic acid method in a sulfuric acid system^21^.

### Statistical analysis

The data were analyzed by one-way ANOVA and performed using the MSTAT-C program (version 2.10). The differences between the treatments’ means were analyzed based on Tukey’s honestly significant difference test (Tukey’s HSD) using the MSTAT-C program at 1 and 5% levels of significance. The Pearson correlation analyses were performed by the SRPLOT program.

## Results

### Available phosphorus

**Fig. 1:**
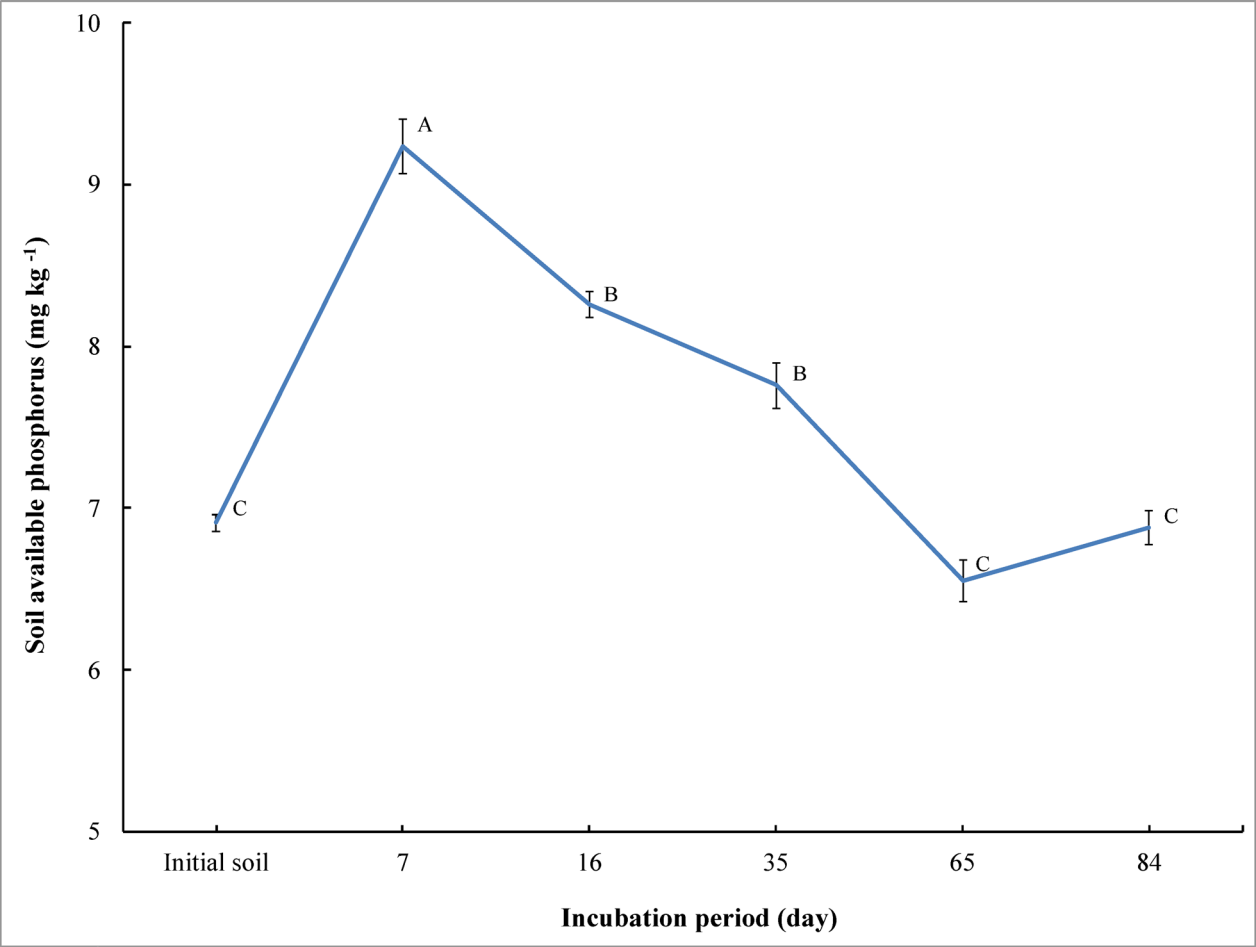
Effect of incubation period on changes of available phosphorus in saline sandy soil under bone char applications. The vertical bars indicate the standard error of the means (*n* = 3). Different letters represent that the means significantly differ according to Tukey’s honestly significant difference test (Tukey’s HSD) at *p* ≤ 0.01.

A significant increase was observed (*p* ≤ 0.01) in available phosphorus with the application of bone char in saline soil after 7, 16, and 35 days of incubation compared to the initial soil (before the incubation and unamended). Available phosphorus increased from 6.91 mg kg^− 1^ for the initial soil to 9.24, 8.26, and 7.76 mg kg^− 1^ at 7, 16, and 35 days, respectively (Fig. 1). Relative to the initial soil, the concentration of available phosphorus increased by 33.7%, 19.5%, and 12.3% after 7, 16, and 35 days, respectively. The results showed that increasing the incubation time significantly decreased phosphorus availability in saline soil when bone char was added. Available phosphorus decreased from 9.24 mg kg^− 1^ at day 7 of incubation to 8.26, 7.76, 6.55, and 6.88 mg kg^− 1^ at 16, 35, 65, and 84 days of incubation, respectively. The highest phosphorus concentration was observed in the saline sandy soil on the seventh day of incubation. Phosphorus availability then decreased with prolongation of the incubation period until reaching its lowest concentration at 65 days of incubation, before increasing again at 84 days of incubation (Fig. 1).

### Distribution of inorganic phosphorus fractions

Significant increase (*p* ≤ 0.01) in NaHCO_3_-Pi, HCl-Pi, and Res-Pi fractions with applying bone char in the soil under study at all incubation periods compared to the initial soil (before the incubation and unamended). On the other hand, applying bone char to saline soil resulted in a significant decrease in the NH_4_Cl-Pi fraction compared to the initial soil. NaOH-Pi fraction shows a non-significant decrease with the addition of bone char (Table 3). The content of NH_4_Cl-Pi fraction decreased after applying bone char from 6.50 mg kg^− 1^ (initial soil before the incubation) to 5.33, 5.31, 5.42, 5.07, and 5.00 mg kg^− 1^ for 7, 16, 35, 65, and 84 days of the incubation period, respectively. Adding bone char to saline sandy soil increased the concentration of NaHCO_3_-Pi fraction from 17.13 mg kg^− 1^ (initial soil before the incubation) to 31.54, 33.39, 35.01, 32.94, and 33.28 mg kg^− 1^ for 7, 16, 35, 65, and 84 days of the incubation period, respectively. The highest NaHCO_3_-Pi fraction content was recorded at 35 days of incubation. The HCl-Pi fraction content increased from 233.25 mg kg^− 1^ (initial soil before the incubation) to 758.25, 743.14, 756.62, 753.95, and 751.23 mg kg^− 1^ for 7, 16, 35, 65, and 84 days of the incubation period, respectively. The residual P fraction increased from 55.79 mg kg^− 1^ for the initial soil before the incubation to 84.81, 91.39, 83.93, 85.61, and 90.26 mg kg^− 1^ for 7, 16, 35, 65, and 84 days of the incubation period, respectively (Table 3). The contents of inorganic phosphorus fractions before and after bone char application to saline sandy soil followed the order of HCl-Pi > Res-P > NaHCO3-Pi > NaOH-Pi ˃ NH_4_Cl-Pi. As for the effect of incubation periods, there was no significant effect on all phosphorus fractions in the soil under study (Table 3).

**Table 3:** Effects of incubation periods on inorganic phosphorus fractions under bone char applications in saline sandy soil. All data were reported as means ± standard error (*n* = 3).

Incubation periods (day)	The concentration of phosphorus fractions (mg kg^− 1^)				
	NH_4_Cl-Pi	NaHCO_3_-Pi	NaOH-Pi	HCl-Pi	Residual *P*
Intial soil	6.50 ± 0.03 A	17.31 ± 0.04B	10.16 ± 0.74 A	233.25 ± 0.50B	55.79 ± 0.12B
7	5.33 ± 0.08B	31.54 ± 0.41 A	8.42 ± 0.08 A	758.25 ± 19.68 A	84.81 ± 1.70 A
16	5.31 ± 0.12B	33.39 ± 0.47 A	9.86 ± 0.15 A	743.14 ± 5.56 A	91.39 ± 1.90 A
35	5.42 ± 0.20B	35.01 ± 0.66 A	8.76 ± 0.30 A	756.62 ± 2.36 A	83.93 ± 4.16 A
65	5.07 ± 0.10B	32.94 ± 1.76 A	8.65 ± 0.25 A	753.95 ± 23.49 A	85.61 ± 9.35 A
84	5.00 ± 0.05B	33.28 ± 0.75 A	8.73 ± 0.35 A	751.23 ± 6.42 A	90.26 ± 1.37 A

*Pi* inorganic phosphorus. Different letters within the same column indicate that the mean significantly differs according to Tukey’s honestly significant difference test (Tukey’s HSD) at *p* ≤ 0.01. NH_4_Cl-Pi: inorganic phosphorus extracted by 1 M NH_4_Cl; NaHCO_3_-Pi: inorganic phosphorus extracted by 0.5 M NaHCO_3_; NaOH-Pi; inorganic phosphorus extracted by 0.1 M NaOH; HCl-Pi: inorganic phosphorus extracted by 1 M HCl.

### Soluble calcium and magnesium

**Fig. 2:**
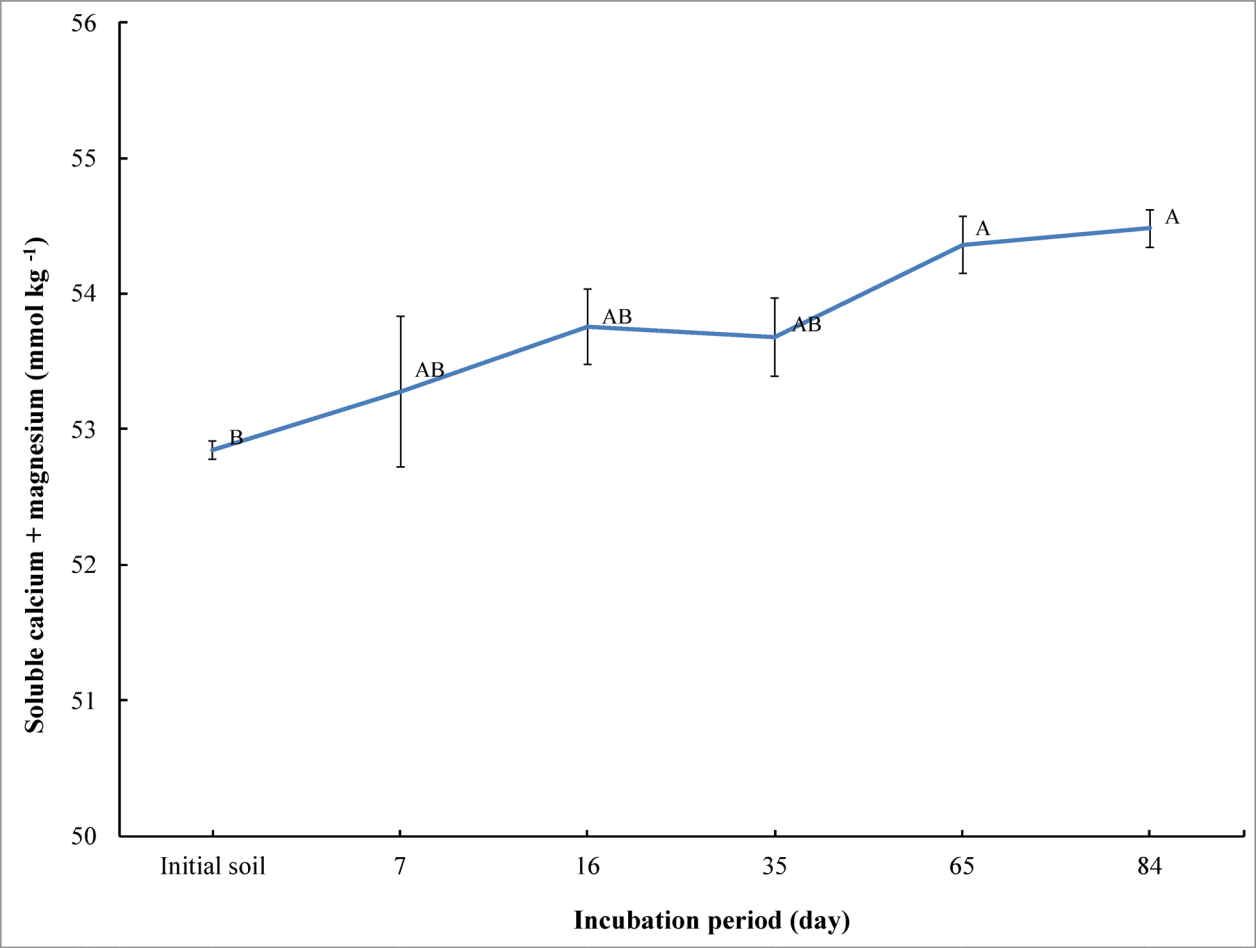
Effect of incubation period on changes of soluble calcium + magnesium in saline sandy soil under bone char applications. The vertical bars indicate the standard error of the means (*n* = 3). Different letters represent that the means significantly differ according to Tukey’s honestly significant difference test (Tukey’s HSD) at *p* ≤ 0.05.

Soluble (calcium + magnesium) significantly increased (*p* ≤ 0.05) upon adding bone char to saline soil at 65 and 84 days of incubation compared to the initial soil (before the incubation and unamended). However, soluble (calcium + magnesium) showed a non-significant increase at 7, 16, and 35 days of incubation, respectively (Fig. 2). Soluble (calcium + magnesium) increased from 52.84 mmol kg^− 1^ for the initial soil to 53.28, 53.76, 53.68, 54.36, and 54.48 mmol kg^− 1^ at 7, 16, 35, 65, and 84 days of incubation, respectively. The highest concentration of soluble (calcium + magnesium) was observed in the saline sandy soil at the end of incubation. But the lowest sulfate concentration was observed in the saline sandy soil before incubation (Fig. 2).

### Soluble sulfate

**Fig. 3:**
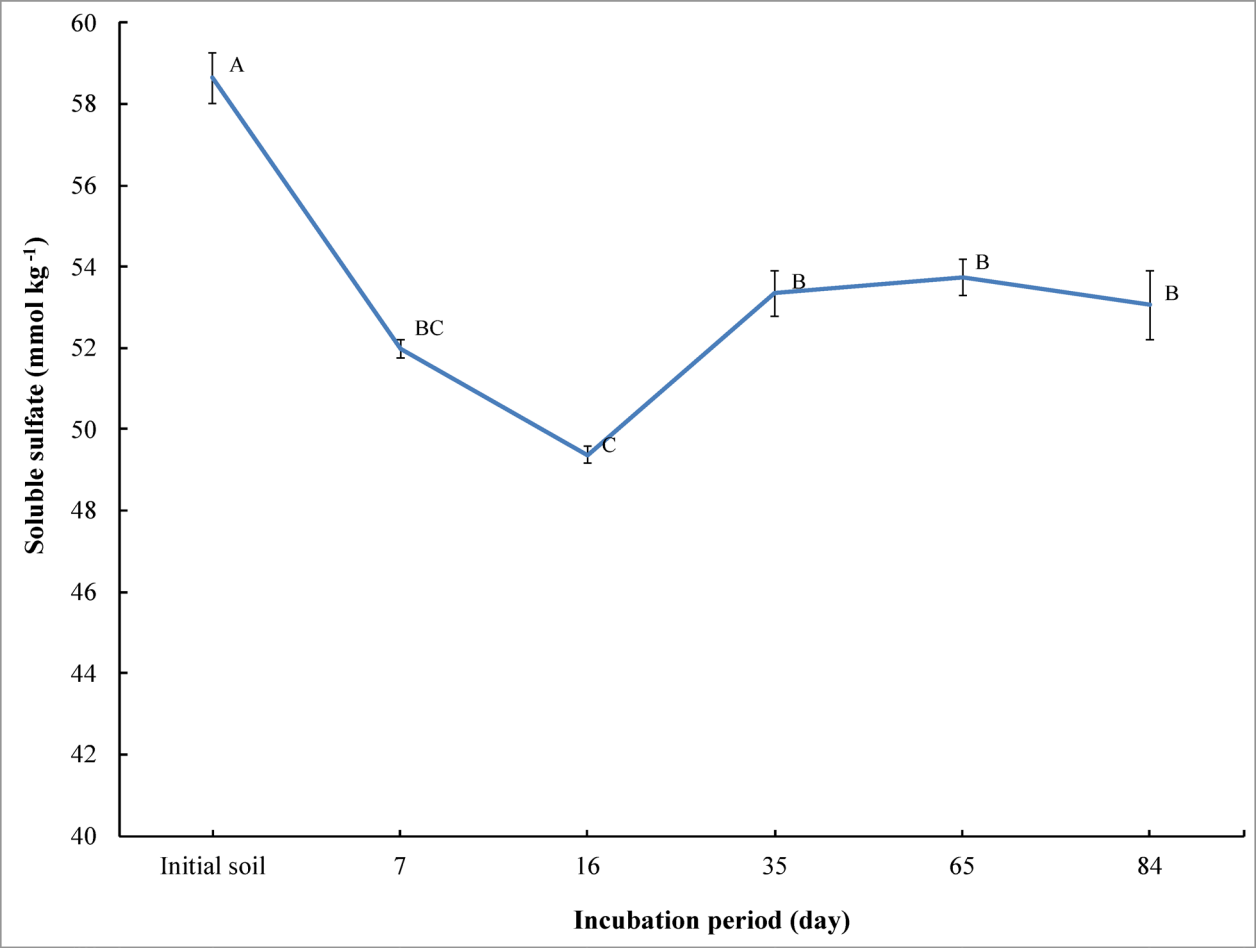
Effect of incubation period on changes of soluble sulfate in saline sandy soil under bone char applications. The vertical bars indicate the standard error of the means (*n* = 3). Different letters represent that the means significantly differ according to Tukey’s honestly significant difference test (Tukey’s HSD) at *p* ≤ 0.01.

The results reported in Fig. 3 show the application of bone char in saline soil on soluble sulfate during different incubation periods. Soluble sulfate significantly decreased (*p* ≤ 0.01) upon adding bone char to saline soil at all incubation periods compared to the initial soil (before the incubation and unamended). Soluble sulfate decreased from 58.64 mmol kg^− 1^ for the initial soil to 51.98, 49.37, 53.34, 53.73, and 53.05 mmol kg^− 1^ at 16, 35, 65, and 84 days of incubation, respectively. With the prolongation of incubation time, soluble sulfate increased significantly at 35, 65, and 84 days of incubation compared to 16 days of incubation. Soluble sulfate increased from 49.37 mmol kg^− 1^ (at 16 days of incubation) to 53.34, 53.73, and 53.05 mmol kg^− 1^ at 35, 65, and 84 days of incubation, respectively. The highest soluble sulfate concentration was observed in the saline sandy soil in the initial soil. But the lowest soluble sulfate concentration was observed in the saline sandy soil after 16 days of incubation (Fig. 3).

### Soluble bicarbonate

Significant decrease (*p* ≤ 0.05) in soluble bicarbonate upon adding bone char to saline soil after 65 days of incubation compared to the initial soil (before the incubation and unamended). Soluble bicarbonate decreased from 5.83 mmol kg^− 1^ for the initial soil to 4.88, 3.62, and 5.04 mmol kg^− 1^ at 16, 65, and 84 days of incubation, respectively. With the prolongation of incubation time, soluble bicarbonate significantly decreased at 16, 65, and 84 days of incubation compared to 7 days of incubation. Soluble bicarbonate decreased from 7.09 mmol kg^− 1^ (at 7 days of incubation) to 4.88, 5.83, 3.62, and 5.04 mmol kg^− 1^ at 16, 35, 65, and 84 days of incubation, respectively. The highest soluble bicarbonate concentration was observed in the saline sandy soil at 7 days of incubation. But the lowest soluble sulfate concentration was observed in the saline sandy soil after 65 days of incubation (Table 4).

**Table 4:** Effects of incubation periods on some chemical soil properties under bone Char applications in saline sandy soil. All data were reported as means ± standard error (*n* = 3).

Incubation period (day)	pH	EC dS m^− 1^	Ion concentrations (mmol kg^− 1^ soil)			
			Soluble calcium	Soluble sodium	Soluble HCO_3_^−^	Soluble Cl^−^
Initial soil	8.05 ± 0.01 A	7.07 ± 0.02 A	46.50 ± 0.14 A	108.03 ± 0.89 A	5.83 ± 0.64AB	122.55 ± 0.82AB
7	8.06 ± 0.01 A	7.12 ± 0.01 A	46.50 ± 0.25 A	109.92 ± 0.28 A	7.09 ± 0.09 A	123.50 ± 0.47 A
16	8.08 ± 0.01 A	6.98 ± 0.02 A	46.67 ± 0.22 A	108.03 ± 0.29 A	4.88 ± 0.64BC	119.23 ± 0.47BC
35	8.06 ± 0.00 A	7.08 ± 0.04 A	46.67 ± 0.30 A	111.03 ± 0.81 A	5.83 ± 0.27AB	123.50 ± 1.26 A
65	8.06 ± 0.01 A	7.04 ± 0.04 A	46.88 ± 0.07 A	108.98 ± 0.98 A	3.62 ± 0.09 C	120.41 ± 0.41ABC
84	8.06 ± 0.01 A	7.03 ± 0.05 A	46.88 ± 0.07 A	109.14 ± 0.35 A	5.04 ± 0.18BC	118.99 ± 0.41 C

Different letters within the same column indicate that the mean significantly differs according to Tukey’s honestly significant difference test (Tukey’s HSD) at *p* ≤ 0.05.

### Soluble chloride

After adding bone char, soluble chloride decreased significantly (*p* ≤ 0.05) in this soil at 84 days of incubation compared to the initial soil. Soluble chloride decreased from 122.55 mmol kg^− 1^ (initial soil) to 119.23, 120.41, and 118.99 mmol kg^− 1^ for 16, 65, and 84 days of incubation, respectively (Table 4). Compared to 7 days of incubation, adding bone char led to a significant decrease in soluble chloride (*p* ≤ 0.05) in this soil at 16 and 84 days of incubation. Overall, the concentration of soluble chloride decreased significantly with increasing incubation periods after the addition of bone char in saline sandy soil (Table 4).

### Some other soil chemical properties

Non-significant effects were observed after adding bone char to saline soil under different incubation periods on pH, electrical conductivity, soluble calcium, and soluble sodium (Table 4).

### The relationships between available phosphorus and phosphorus fractions with some soil chemical properties

**Fig. 4:**
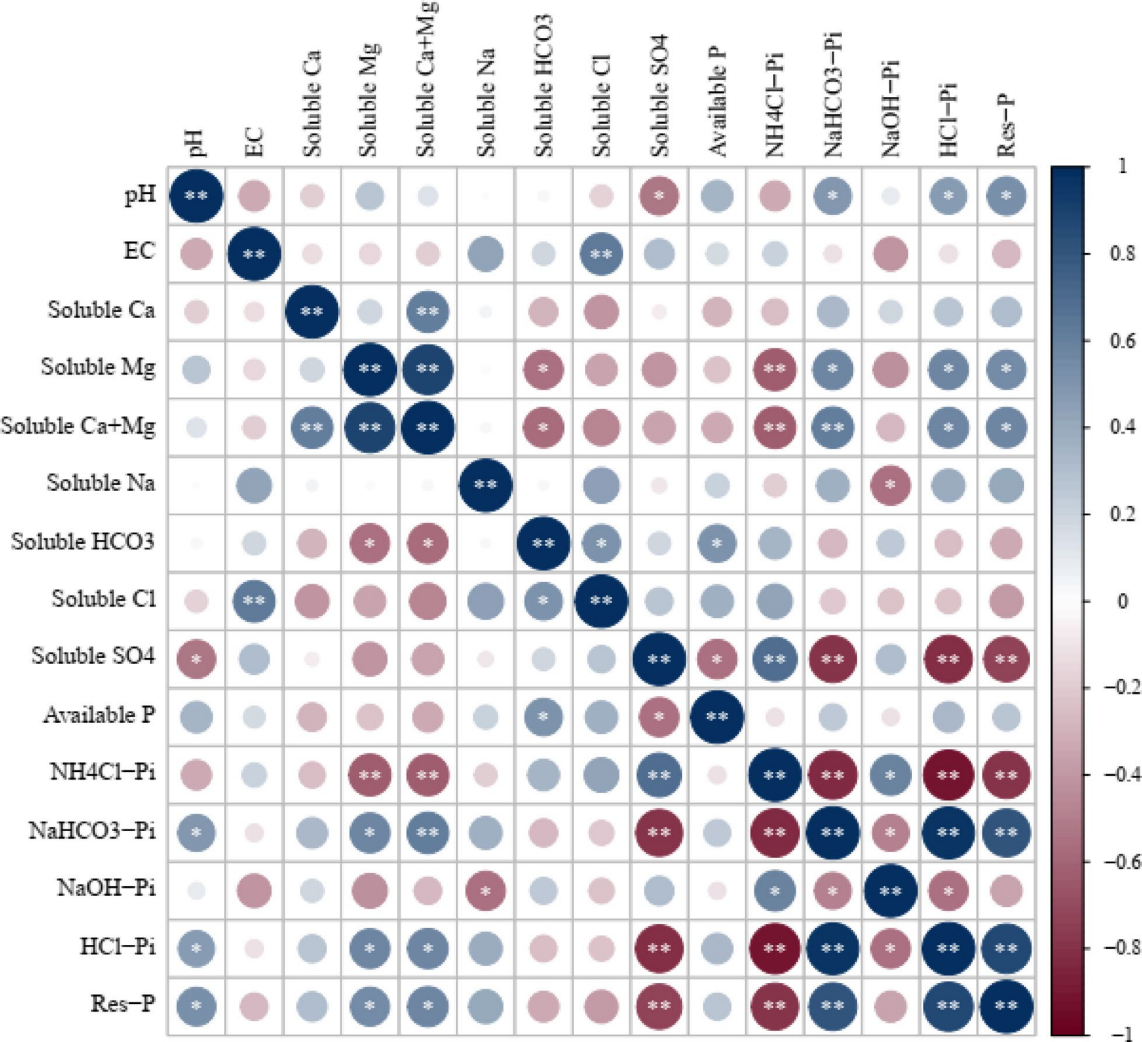
Pearson correlation matrix featuring relationships between available phosphorus and phosphorus fractions with some soil properties in saline sandy soil. In the correlation analysis diagram, blue color represents a positive correlation and red color represents a negative correlation. **Correlation is significant at *p* ≤ 0.01; *correlation is significant at *p* ≤ 0.05.

The Pearson correlation matrix obtained from this study is shown in Fig. 4. The correlation coefficient between available phosphorus and soluble HCO_3_^−^ was positive and significant. But, the correlation coefficient between available phosphorus and soluble SO_4_^2−^ was negative and significant. The correlation coefficient between NH_4_Cl-Pi fraction and soluble SO_4_^2−^ was positive and highly significant, as well as the correlation coefficient between NH_4_Cl-Pi fraction and NaOH-Pi was positive and significant. However, the NH_4_Cl-Pi fraction negatively correlated with soluble Mg, soluble Mg + Ca, NaHCO_3_-Pi, HCl-Pi, and Res-P, and this correlation was highly significant. The results showed that NaHCO_3_-Pi had a highly significant positive relationship with soluble Mg + Ca, HCl-Pi, and Res-P, as well as a significant positive relationship with pH and soluble Mg, while NaHCO_3_-Pi fraction had a highly significant negative correlation with soluble SO_4_^2−^ as well as a significant negative correlation with NaOH-Pi fraction. A significant negative correlation was found between NaOH-Pi fraction with soluble sodium and HCl-Pi fraction. A highly significant positive correlation was found between the HCl-Pi fraction with Res-P, as well as the correlation coefficients between the HCl-Pi fraction with pH, soluble Mg, and soluble Mg + Ca were positive and significant. But, the correlation coefficient between HCl-Pi fraction and soluble SO_4_^2−^ was negative and highly significant. The relationships between the Res-P fraction with pH, soluble Mg, and soluble Mg + Ca were positive correlations and significant. However, the correlation coefficient between Res-P fraction and soluble SO_4_^2−^ was negative and highly significant.

## Discussion

The availability of phosphorus in soils relies on several main factors, including mineralogy composition, acidity, element concentrations in the soil solution, and sources of phosphate fertilizers^28^. Moreover, the concentration of available phosphorus in the soil was highly dependent on the phosphorus fertilizer amount and the incubation time^29^. The results obtained from this study were compatible with several studies, which found that the addition of bone char to many soils differs in their chemical properties, causing a significant increase in phosphorus availability. This is attributed to the dissolution of phosphorus from bone char because it contains high phosphorus content^20,30,31^. In the present study, phosphorus availability in saline soil decreased significantly with increasing incubation time. This result was in line with Akhtar and Alam^32^ who found that phosphorus availability in soil gradually decreased with increasing incubation time because of increased phosphorus sorption. The rate of available phosphorus release in sandy soil was very high in the first few days of incubation, then it continued more slowly until an apparent equilibrium was approached^33^. One reason for the low solubility of phosphorus is attributed to the formation of insoluble calcium phosphate compounds is the presence of dissolved calcium in the soil solution at high concentrations^34^. On the other hand, many studies found that increasing phosphorus availability with prolonged incubation time under bone char applications in some soils^35,36^. Phosphorus precipitation as calcium phosphate form is greatly dependent on the Ca/P ratio at constant pH^37^. The concentrations of H^+^, Ca^2+^, and H_2_PO_4_^−^ in the soil solution play an important role in greatly affecting phosphorus dissolution from bone char in different types of soils^20^. Calcium phosphate dissolution from bone char led to the release of Ca and H_2_PO_4_^−^ ions into the soil solution; therefore, the reaction will not proceed if soluble Ca and H_2_PO_4_^−^ concentrations rise too far. Sink size for dissolved phosphorus was the other significant control on the dissolution of bone char^20^. Generally, the precipitation–dissolution reactions of phosphorus in alkaline soils are highly dependent on soil pH, calcium concentrations, and bicarbonate and sulfate concentrations^38^. Moreover, insoluble calcium phosphate compounds are formed in some soils when rise calcium activity and pH^39^. Björkman and Reiners^40^ found that the presence of bicarbonate ions improved phosphorus availability in sandy soil because of bicarbonate that desorbs phosphate from solid-phase binding sites.

Using phosphorus fractionation, we can determine the nature, solubility, and relative bioavailability of phosphorus. A sequential extraction of phosphorus was utilized to determine the distribution of plant-available phosphorus fractions in soils^41^. Phosphorus fractions in soils are divided into four groups: (1) labile P includes NH_4_Cl-Pi and NaHCO_3_-Pi, (2) moderately labile P contains NaOH-Pi, (3) moderately stable P is HCl-Pi, and (4) stable P or non-labile is residual P^42,43^. The NH_4_Cl-Pi fraction is expressed as soluble phosphorus and loosely bound to the exchange sites; this phosphorus is considered available for plant uptake^44^. The NaHCO_3_-Pi fraction is expressed as adsorbed on the soil surfaces, labile, and available for plants^45^. NaOH-Pi fraction expresses the inorganic phosphorus associated strongly with chemisorption on the surfaces of aluminum and iron oxides and carbonates^46,47^. The HCl-Pi fraction expresses occluded and slow-turnover inorganic phosphorus associated with calcium, which is present in apatite or octacalcium phosphate^45,46^. Residual P is non-labile; stable organic phosphorus forms and relatively insoluble inorganic forms^46^. The concentration and proportions of the different phosphorus fractions depend on the soil type and management. These fractions exhibit significant differences in their mobility, bioavailability, and chemical behavior in the soil, and can be transformed under certain soil conditions^47^. Salinity plays an important role in phosphorus fractionation and its availability in soil, which in turn affects crop growth and yields^12^. The results obtained from this study were compatible with Amin^30^who found that adding bone char to some soils caused a significant increase in the NaHCO_3_-Pi, HCl-Pi, and residual P fraction compared to the unamended soil. Also, the bone char application caused significant decreases in NH_4_Cl-Pi fraction in soil compared to the unamended soil. In several studies, adding different sources of phosphate fertilizers did not significantly affect the NaOH-Pi fraction^48,49^. The increase in NaHCO_3_-Pi fraction may be attributed to the bone char used in this study has a high content of available phosphorus. This effect was compatible with the results of Matelele et al.^50^ who found that there is a significant positive correlation between NaHCO_3_-Pi fraction with HCl-Pi. The highest content of P fractions was observed as the insoluble P associated with calcium, resulting from bone char applications in soils^48,51^. HCl-Pi recorded the highest content of all inorganic phosphorus fractions is attributed to the phosphorus presence in some forms like octacalcium phosphate and hydroxyapatite^52^. This is attributed to the high content of apatite in the bone char material^53^. Generally, a high content of basic cations in soil solution causes an increase in moderately labile and non-labile P fractions^28^. The correlation between HCl-P fraction and soil available phosphorus was positive. This indicates that Ca-bound P plays a role in removing labile P from the soil solution via an adsorption or precipitation mechanism that may be observed in the correlation between available phosphorus and HCl-P fraction^47^. This result appears to agree with Azene et al.^54^ who suggested that the soil pH has a strong positive correlation with the HCl-Pi fraction. This is attributed to soil commonly has high calcium content at high soil pH and the existing phosphorus in the soil precipitates with Ca. There is also a positive relationship between HCl-Pi fraction and calcium content was strongly positively correlated. This finding was in agreement with the results from a previous study by Matelele et al.^50^ who emphasized that there is a significant positive correlation between HCl-Pi fraction with NaHCO_3_-Pi and Res-P.

Our results found that soluble (calcium + magnesium) increased under applying bone char because of the high concentrations of cations such as Ca and Mg from the bone char^31,55^. The lower amounts of sulfate, as observed in the soil after incubation, might be attributed to the formation of insoluble calcium compounds like gypsum^56^. Generally, sulfate and chloride ions were important factors that had an impact on releasing phosphorus from the sediment^57^. On the other hand, the concentrations of available phosphorus in some alkaline soils were positively correlated with soluble sulfate^30^.

We have conducted numerous studies examining the behavior of bone char in various alkaline soils with different chemical properties. In addition to this study, we tested phosphorus release from bone char in saline soils. To explain the environmental factors and soil properties affecting the dissolution of phosphorus from bone char in all alkaline soils. Several studies have suggested that using bone char is a promising strategy and alternative to phosphate fertilizers in sustainable agriculture. This is attributed to its high phosphorus content, its renewable resource, and its ability to avoid soil contamination by heavy metals and radionuclides^58,59^. The phosphorus utilization from bone char in cultivation can be similar to a soluble phosphorus source under some conditions^55^. Using slow-release phosphate fertilizers is one of the strategies to increase phosphorus availability, which is more favorable than using soluble phosphate fertilizers in alkaline soils because of the increased phosphorus in soil solution, resulting in the formation of insoluble calcium phosphate^60^. Utilizing bone char as a slow-release fertilizer is advantageous because it reduces the risk of over-fertilization and nutrient leaching, which negatively affects the ecosystem^61^. Bone char application as a soil amendment reduces dependence on conventional chemical phosphate fertilizers derived from rock phosphate, which helps achieve sustainable development goals and a circular economy^15^.

On the other hand, several limitations face bone char applications in different soils as a fertilizer, such as the very low solubility of phosphorus compounds in bone char, as it mostly exists in the hydroxyapatite form, preparation methods affecting several conditions, such as pyrolysis temperature, heating rate, residence time, and animal bone type. These factors influence the physical and chemical properties of the produced bone char. Production quantity of bone char greatly depends on the availability of an energy source, the amount of bones, bone collection costs, and the method of preparation. Soil properties play a pivotal role in influencing the solubility of phosphorus from bone char, especially pH and soluble ions in the soil solution. Application doses of bone char rely on soil type and bone char characteristics. There are many uses for bone char, such as water purification, paints, and purifying sugar during its manufacture; all these compete with using bone char as a fertilizer, which would affect the availability of bone char in the markets. Field experiments on the use of bone char as a fertilizer in soil are very few. Despite all these limitations, the high phosphorus content of bone char is considered a potential source of phosphorus, and more studies should be conducted on the fate of phosphorus under field conditions.

## Conclusions

Converting animal bone into bone char represents a kind of recycling of phosphorus from bone waste, which is expected to be a promising alternative to chemical phosphate fertilizers due to its high phosphorus content and being a renewable source. Therefore, this study aims to evaluate the effect of the incubation period on changes in some chemical soil properties and the availability and fractionation of phosphorus in saline sandy soil by applying bone char. At the beginning of the bone char addition, the concentration of available phosphorus in saline sandy soil was high. Ionic compositions in the soil solution in saline sandy soils may play an important role in influencing the release of available phosphorus from bone char. Therefore, further research on agricultural practices for improving phosphorus release from bone char should be encouraged. Investigating the distribution of phosphorus fractions in soil is important for comprehending phosphorus behavior, its availability to plants, and its impact on soil productivity. The future stage will move from laboratory studies to more representative field studies on bone char applications in soils. This will help us find an alternative source of chemical phosphate fertilizers, such as bone char, because chemical phosphate fertilizers are produced from non-renewable sources. Using bone char as a soil amendment represents a promising step towards sustainable agriculture and the circular economy, as well as reducing environmental and health risks. Additionally, bone char is a natural source of phosphorus that can be used as a slow-release fertilizer in agriculture. However, the limitations of using bone char as a fertilizer include very low solubility of phosphorus in bone char, preparation methods, production quantity, soil properties, application doses, bone char availability in markets, and lack of field experiments.

## Data Availability

The datasets used or analyzed during the current study are available from the corresponding author upon reasonable request.
